# Economic transformation based on leading commodities through sustainable development of the oil palm industry

**DOI:** 10.1016/j.heliyon.2024.e25674

**Published:** 2024-02-13

**Authors:** Fitri Hariyanti, Almasdi Syahza, Zulkarnain, Nofrizal

**Affiliations:** a Department of Enviromental Science, Graduate Program Riau University, Pekanbaru, Riau, 28127, Indonesia; b Division of Regional Accounts and Statistical Analysis, BPS-Statistics of Riau Province, 12 Pattimura Street, Pekanbaru, Riau, 28131, Indonesia

**Keywords:** Economic transformation, Leading commodity, Sustainable, Prospective analysis, SDGs

## Abstract

The oil palm industry is a strategic and leading sector of agriculture that is developing in Indonesia. Riau is a province in this country that has quite large natural resources in the plantation with the largest contribution being oil palm. This study aims to determine the form of economic transformation based on superior commodities through the development of a sustainable palm oil industry. The study adopts a mixed-method approach, employing a sequential explanatory model to gather a comprehensive understanding of the oil palm industry in Bengkalis Regency, Indonesia. Quantitative data collection precedes qualitative data analysis, allowing for a deeper exploration of the research topic. The analysis units encompass the oil palm industry, households, and village officials, ensuring a holistic examination of the subject. Data collection involves a range of methods, including surveys, interviews, observations, and focus group discussions, engaging diverse stakeholders such as community members, industry representatives, and government officials. Secondary data from relevant agencies and previous research are also integrated. Ethical considerations are emphasized in data collection and dissemination, ensuring transparency and balance in reporting. Overall, the methodology is well-structured and comprehensive, enabling a thorough examination of the industry's sustainability and development. The research results indicate that the oil palm manufacturing industry is a leading sector and has been categorized as sufficiently sustainable based on different perspectives. Through an optimistic scenario, the government must make efforts and total support to improve the important factors that influence the development of the palm oil processing industry in a sustainable manner, including the community empowerment program, future palm oil prospects, implementation of environmental management.

## 1 Introduction

The oil palm industry is a strategic and leading sector of agriculture that is well-developed in tropical countries, such as Indonesia, Malaysia, and Thailand. A leading sector as defined by Gaveau, is an industry that is currently playing a major role in a region's economic development based on its advantages and criteria. The four criteria of a leading sector include 1) high economic growth, 2) relatively high labor absorption rate, 3) high linkages between sectors both forward and backward, and 4) creation of high added value [1]. Riau is a province in Indonesia that has large natural resources, such as oil and oil palm plantation. The reduced oil production since 2013 has led to a shift in Riau's economic structure from the mining dominance to the agricultural/plantation and industrial sector. Previous research, found that oil palm has the biggest contribution among several other plantation commodities, such as coconut and rubber [2].

The development and production of the crude palm oil (CPO) manufacturing industry and its derivatives follow the plantation area and the palm production growth as a raw material [3]. Previous research, the oil palm plantation expansion was followed by an increase in CPO production from 1.79 million tons in 2001 to 7.57 million tons in 2013, amounting to 12.76% average growth [4]. Riau has great potential to promote oil-derived products, thereby becoming a leading and priority plantation commodity in the future [5].

The development of Riau's oil palm plantation and industry has a double impact on the regional economy, particularly through the creation of more jobs. In general it can be said that with the existence of the area oil palm plantations have managed to improve income of oil palm farming communities in rural areas [4]. Previous research results showed that the average income of smallholders in the plantation sub-sector (especially oil palm) was US$ 481.75 per month or US$ 5781.09 per year. This value is far above the 2019 Riau Provincial Minimum Wage (UMP) limit of US$ 190.15 [6]. This makes a significant contribution to the national economy for producing countries and provides benefits to the local economy [7]. Investment in the palm oil industry has an impact on increasing socio-economic development in rural areas. This aspect is also related to the value of benefits for society and the environment [8]. The impact of investments in the plantation sub-sector has been felt by rural communities, particularly regarding people's purchasing power. This has led to an increase in goods and people mobility.

The oil palm plantation industry in Indonesia is faced with various sustainability issues that can hinder trade access in the global market [9]. Currently, the market demand for CPO continues to increase, which means the oil palm industry's potential is growing continuously but not directly proportional to the environmental management quality. Previous research, discovered that 1 ton of oil palm produces waste in the form of empty fruit bunches amounting to 23% or 230 kg, 6.5% or 65 kg shell, 4% or 40 kg wet decanter solid (palm mud), 13% or 130 kg fiber, and 50% liquid [[10], [11], [12]]. All waste from palm oil derivatives can be reused, especially for organic fertilizer, animal feed and a source of protein for livestock [7]. Environmental degradation due to the use of fertilizers and chemicals pollutes river water and air, reduces the quantity of groundwater, and causes soil erosion [13].

In accordance with sustainable development goals (SDGs), the government issued policies related to oil palm development. In this scenario, ecological sustainability was considered because economic and societal sustainability depends on the biosphere and ecological processes' integrity. It emphasizes that economic sustainability improves human well-being quality, while the social involves people's equal opportunity and ability to overcome the main problems in their lives [14].

It is important to note that the CPO industry sustainability has complexities and complicated problems. An example is environmental issues, which involve the belief in the benefits of economic, social, cultural, and other aspects. This becomes the urgency and justification for the need to deeply and objectively explore the oil palm industry's sustainability. This study aims to determine the form of economic transformation based on superior commodities through the development of a sustainable palm oil industry. The benefits of this research study can be a reference for developing a sustainable palm oil industry system to achieve sustainable development goals.

## 2 Methodology

### 2.1 Study approach and method

A mixed-method approach was used in this study's design, in which the quantitative technique was used as the opening data to strengthen the informational data obtained qualitatively [15]. In this method, a sequential explanatory model was utilized in the first stage by collecting and analyzing quantitative data. In the second phase, data collection and qualitative data analysis were performed. Subsequently, the overall data were analyzed and conclusions were drawn [16,17].

### 2.2 Study design and sample

This study is conducted in Bengkalis Regency. The analysis unit includes the oil palm industry, while the participants involved directly and indirectly were households and village officials. Based on the first unit of analysis, the total population is 14 companies. The second unit of analysis is households living around the industry. These households were selected purposively, resulting in a sample size of 400 households. This sample size is feasible to use in the study [16]. The third unit of analysis is the village area where the palm oil processing industry is located.

### 2.3 Data collection

In this study, primary and secondary data were collected and analyzed. The primary data were extracted directly through interviews, surveys, and observations. Furthermore, the secondary data were sourced from related agencies, such as the Central Statistics Agency, the Environment and Forestry Office of Riau Province, the Ministry of Environment and Forestry, the Industry Office of Riau Province, the Regional Development Planning Agency for Research and Development of Riau Province, and the other related study's results in the form of articles, journals, or reports from relevant agencies.

The data collection techniques employed include 1) Survey, which was conducted by distributing questionnaires to obtain quantitative data. Questionnaires were distributed to respondents in order to answer questions related to the study topic. 2) Interview, which emphasized the in-depth discussions with the participants who came from the communities around the oil palm industry, managers and workers, heads and village officials around the place, regional/city government, and Non-Government Organizations (NGOs) related to the industry and its environment. 3) Observation, which serves as a step for examining the activities or events occurring directly during the study process. Furthermore, this technique was used to explore data that strengthened quantitative and qualitative results, for example by photographing the oil palm industry process using the camera. This makes the study object visualization to be more real, objective, and factual. 4) Focus Group Discussion (FGD) was conducted to clarify results and analyze data useful as inputs in preparing models and scenarios for the sustainable regional development of the oil palm industry. In addition, the FGD technique is an “ethics” manifestation and dissemination, in which its results are presented and reported in a balanced and objective manner. The FGD was conducted with 25 participants from institutions involved in the development of the industry, such as the Plantation Agency, Regional Development Planning Agency, Environment and Forestry Agency, Central Bureau of Statistics, Palm Oil Association, Researchers, and others. All respondents in data collection, whether through surveys, interviews and FGDs, were well-informed about their contribution to this research. Likewise, they gave consent to use the data for research purposes.

### 2.4 Data analysis

A. Analysis of Leading Commodity-based Economic Transformation

Quantitative descriptive analysis is used to analyze the results of calculating the transformation of the economic structure, namely using Production Structure Analysis. As well as using Shift Share analysis and Growth Ratio Model (MRP) to find out the leading sectors and commodities of a region. Analysis of Production Structure and Labor Allocation, namely by comparing the relative shares of the sectors of the production structure and labor allocation If classified, the production sector is divided into 3 groups, namely: Primary Sector, namely the production sector which does not process raw materials, but only takes and utilizes natural resources directly such as land and deposits therein. For example the agricultural and mining and quarrying sectors; Secondary sector, namely the production sector that processes raw materials, both from the primary sector and from the secondary sector itself. Converting semi-finished goods into other goods with a higher value, for example the manufacturing sector and the construction sector; and Tertiary sector, namely sectors that do not produce in physical form but in the form of services, for example the trade sector, banks and financial institutions, housing rental, government and services. The utility sector is also included in the tertiary sector, covering the electricity, gas and drinking water, as well as transportation and communication sectors.

The Shift share analysis is a model typically used to observe the regional growth patterns and rates needed to be realized when the regional productivity is compared with the national (formula 1). Furthermore, the Shift component in the shift-share analysis showed the variances occurring in the share component value due to the application of sector-specific factors and the local issues affecting the economy concerned [18]. The four parts considered in this method include.

a ***Regional Share* (RS)** is a component of regional economic growth caused by external factors. Furthermore, RS showed an increase in regional economic activities due to the prevailing national policies.
b ***Proportional Shift* (PS)** is a regional economic growth component caused by a good economic structure, particularly in fast-growing sectors.
c ***Differential Shift* (DS)** is caused by competitive regional-specific conditions that promoted regional export growth.
d ***Shift Share* (SS)** is the sum of Regional Share with Proportional and Differential Share.

These four elements are formulated below in order to examine the advantages of the area

(1) $${RS}_{ij}=y_{ijo}\;\left(\frac{y_{t}}{y_{o}}\;‐1\right)\;{PS}_{ij}=y_{ijo}\;\left(\frac{y_{it}}{y_{io}}\;-\;\frac{y_{t}}{y_{o}}\;\right)\;{DS}_{ij}=y_{ijo}\;\left(\frac{y_{ijt}}{y_{ijo}}\;-\;\frac{y_{it}}{y_{io}}\;\right)\;{SS}_{ij}={RS}_{ij}+{PS}_{ij}+{DS}_{ij}$$

Note:

Y_t_ = GDP of the reference area for the final year period.

Y_o_ = GDP of the reference area for the initial year period.

Y_it_ = GDP of the reference area of the i sector for the final year period.

Y_io_ = GDP of the reference area of the i sector for the initial year period.

Y_ijt_ = GRDP of the i-sector analysis area for the final year period.

Y_ijo_ = GRDP of the i-sector analysis area for the initial year period.

The measurement results above were interpreted as follows: (1) When PSij >0, it means the sector i in an analysis area grows faster than that of the reference area, and vice versa. (2) When DSij >0, it indicates the competitiveness of sector i in an analysis area is higher than that of the reference area and vice versa. (3) When SSij >0, then there is an increase in absolute value or an increase in the regional economic performance of sector i in the analysis area.

The growth ratio model (GRM) method is an analytical tool obtained by modifying the Shift Share Analysis model (formula 2). It identifies potential economic sectors based on GRDP growth criteria (competitive advantage) by comparing a sector's growth in a region with a larger area, both on a large and small scale. The two growth ratios calculated in this analysis include the study area growth (RPs) and the reference area (RPr). To examine the leading sector of an island, the formula used is as follows:

(2) $${RP}_{iP}=\frac{\left(y_{ipt}-\;y_{ipo}\right)/{\;y}_{ipt}}{\left(y_{pt}-\;y_{po}\;\right)/\;y_{po}}\;{RP}_{in}=\frac{\left(y_{\mathrm{int}}-\;y_{ino}\right)/{\;y}_{\mathrm{int}}}{\left(y_{nt}-\;y_{no}\;\right)/\;y_{no}}$$

Note:

Y_ipt_ = GRDP of sector i of the p-th analysis area in the final year period.

Y_ip0_ = GRDP of sector i of the p-th analysis area in the initial year period.

Y_pt_ = GRDP of the total area of analysis p in the final year period.

Y_p0_ = GRDP Total of the analysis area p in the initial year period.

Y_int_ = GDP of sector i reference area in the final year period.

Y_in0_ = GDP of sector i reference area in the initial year period.

Y_nt_ = GDP of the reference area in the final year period.

Y_n0_ = GDP of the reference area in the initial year period.

It is important to note that the GRM only considers the sector growth, without observing the sector contribution in an area. The results are interpreted as follows, (1) when the RP_ip_ and RP_in_ values are positive, the growth of sector i in the analysis and reference areas are both high. This simply means the sector has potential both at the regional and global levels. (2) When the respective RP_ip_ and RP_in_ values are positive and negative, then the growth of sector i in the analysis area is higher than the reference area. This also indicates that the sector has potential at the regional level but has none globally. (3) When the RP_ip_ value is negative and the RP_in_is positive, then the growth of sector i in the analysis area is lower compared to that of reference. It implies the sector has potential at the global level but has no potential regionally. (4) When both RP_ip_ and RP_in_ values are negative, then sector i growth in the analysis and reference areas are both low. This means that the sector has no potential at both the regional and global levels (reference area).

B. The Sustainability Status Analysis of the Oil Palm Manufacturing Industry

The index and sustainability status analysis was performed using the modified RAP-CPO ordinance technique from RAP-Fish. This sets things in a measurable order through the Multi-Dimensional Scaling (MDS) method in order to assess the CPO industry's index and sustainability status. It was observed that the MDS approach in the RAP-FISH produced more stable results than other multivariable analysis methods [19,20]. In this study, the RAP-CPO ordinance analysis using the MDS method was conducted in several stages as follows.

(1) Attributes determination: There are 58 attributes, which are categorized into 5 dimensions, including 17 ecologies, 13 economics, 11 socio-cultural, 7 institutions, and 10 technologies.
(2) Assessment of each attribute on an ordinal scale (scoring) based on each dimension's sustainability criteria.
(3) RAP-CPO ordinance analysis with the MDS method using the SPSS/Excel program to determine the ordinance and stress value.
(4) The sustainability index and CPO's status assessment, which is reviewed as single and multi-dimensions.
(5) Sensitivity Analysis (Leverage Analysis) to determine sensitive variables affecting sustainability.
(6) Monte Carlo analysis to calculate the uncertainty aspects [19,21].

In MDS, two similar objects are mapped at adjacent points, while those that differ are represented by distant points. Each variable's score formed a matrix, which is then standardized for their respective sores (formula 3). This makes each variable have a similar weight, while the differences between measurements are eliminated. The standardization method used is as follows:

[3] $$X_{\text{ik}}\;\text{sd}=\frac{X_{\text{ik}}{\;}_{–}\;X}{S_{k}}$$

Note:

X_ik_ sd = The area standard score (including the reference point) i-th = 1, 2, …n, for each k-th = 1, 2, …p attribute;

X_ik_ = The area initial score (including the reference points) i-th = 1, 2, …n, for each k-th = 1, 2, …p attribute;

X_k_ = The mean score for each kth = 1, 2, …p attribute;

S_k_ = The standard deviation of scores on each kth = 1, 2, …p attribute.

C. Strategy Analysis of the Sustainable Oil Palm Manufacturing Industry

The prospective analysis in this study is used to determine the dominant variables affecting the CPO industry. The Participatory Prospective Analysis (PPA) method is a tool designed for investigating and anticipating changes with expert participation, including stakeholders, which provides quick results. It is important to note that this method can be conducted with a broader approach developed by CIRAD and CAPSA. There are eight stages in the developed method, which include: 1) Definition of the system boundaries, 2) Variables Identification, 3) Key variables definition 4) Mutual influence analysis, 5) Interpretation of the relationship between the influence and dependence, 6) Sate variables’ definition, 7) Scenarios development, and 8) Strategy implications and anticipatory steps [22].

The matrix shown in Table 1 described the inter-factors direct influence on the system performed in the first stage of the prospective analysis. Experts or stakeholders were directly involved when determining the direct influence between factors by entering the scores in the matrix from 0 to 3.

**Table 1:** Inter-factors' direct effects on the sustainable development of the CPO industry.

From↓	A	B	C	D	E	F	G	H	I	J
To→										
**A**										
**B**										
**C**										
**D**										
**E**										
**F**										
**G**										
**H**										
**I**										
**J**										

Source [5,23].

Description: A-J = Factor important in the system.

Score: 0: Noleverage; 1: Little effect; 2: Moderate effect; 3: Very strong effect.

**Fig. 1:**
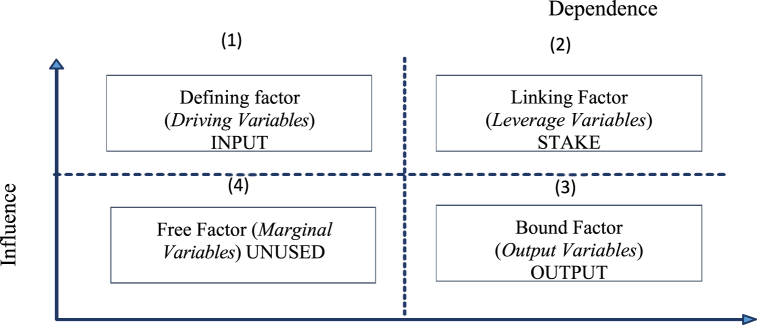
Matrix of inter-factor influence and dependence.

A prospective analysis program was used to determine the key/dominant factors showing the level of influence and dependency between factors in the system, with the representation presented in Fig. 1 [22,24]. According to Bourgeois and Jesus, each quadrant in the diagram has different factor characteristics, such as 1) The first quadrant *(driving variables)*, which contained factors having strong influence but less strong dependence. Furthermore, this quadrant's factors are the driving variables included in the system's strongest category in system. 2) Quadrant two has factors indicating a strong influence and dependence among others *(leverage variables)*. Some of the factors in this quadrant are considered strong variables. 3) Quadrant three *(output variables)* has factors representing output variables, in which the effect is minimal but the dependence is strong. 4) Quadrant four *(marginal variables)* contained marginal variables, which have little effect and low dependence, hence, the factor is independent of the system [16].

## 3 Result and discussion

### 3.1 Leading Commodity-based economic transformation

**Fig. 2:**
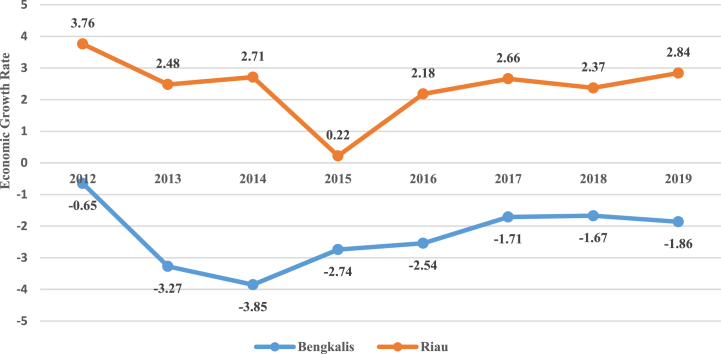
Oil and gas economic growth rate in Bengkalis Regency and Riau Province in 2012–2019.

Bengkalis Regency is one of the regencies in Riau Province. Since 2012 Bengkalis's economy has continued to decline. This was marked by the contraction of Bengkalis' economic growth which reached minus 0.65 percent and reached its peak in 2014 reaching minus 3.85. The main cause of the decline in Bengkalis's economy is due to the natural declining of oil wells in Bengkalis so that oil production continues to decline (Fig. 2).

**Fig. 3:**
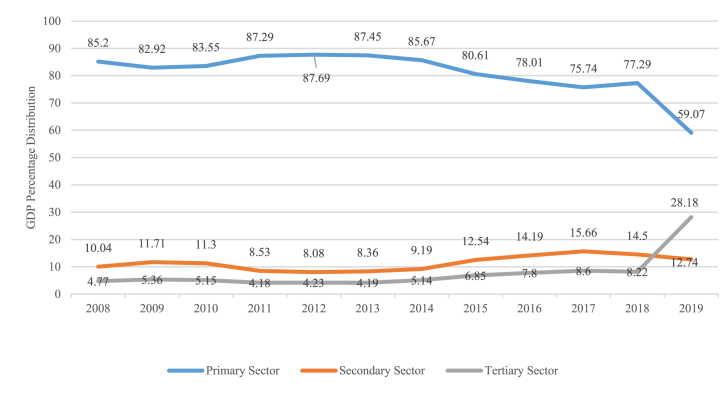
Transformation of the economic structure of Bengkalis Regency based on GDRP, 2008–2019.

With the diminishing contribution of the mining sector, it will increase the contribution of other economic sectors. These changes will result in changes to the economic structure. Based on Fig. 3, in 2008 the distribution of the primary sector was 85.20 percent and decreased to 59.07 percent in 2019. On the other hand, the relative share of the secondary sector increased from 10.04 percent in 2008 to 12.74 percent in 2019. The relative share of the primary sector tends to decrease and the relative share of the secondary sector tends to increase. These conditions indicate that Bengkalis is experiencing a transformation of its economic structure from the primary sector to the secondary and tertiary sectors.

Shift-share analysis is a technique for identifying several factors influencing differences in economic growth and performance that exist in various regions. Furthermore, the Shift Share analysis was used to observe the shift and economic role of a region [25,26]. The results were typically used to 1) describe the relationship between the Bengkalis area and Riau Province, 2) illustrate the economic productivity of Bengkalis, and 3) compare the Bengkalis’ productivity with that of Riau Province. Shift Share analysis is also used to explain the changes in the economic structure occurring in Bengkalis by the growth rate of each business field category (Fig. 3).

It was observed from the Shift Share analysis result for all business field categories shown in Table 2 that the manufacturing industry grows faster and has higher competitiveness than the same category in Riau Province. The manufacturing industry is the category that has the largest proportional growth component from 2008 to 2019. This result is in accordance with the results of previous research that the manufacturing industry is a leading sector, including [27,28]: When viewed from the manufacturing industry subcategory shown in Table 3, the Food and Beverage Companies are the subcategory with the highest advantage. It was also observed that the regional share component indicated by the DS value is the largest among other categories. This means the Food and Beverage Manufacturing Industry has the largest comparative advantage and the potential to be exported to other regions. The development of oil palm will provide great opportunities for the manufacturing industry, especially palm oil derivative products. Furthermore, sustainable development includes harmonious integration between economic growth, environmental preservation, and social conditions [[29], [30], [31]].

**Table 2:** Shift share calculation results.

Category	Description	Shift Share (million IDR)			
		RS	PS	DS	SS
A	Agriculture, Forestry, and Fisheries	4,302,642.3	−1,141,355.9	43,223.3	3,204,509.7
B	Mining and excavation	5,610,612.9	−11,669,743.9	9,849,084.6	3,789,953.6
C	Manufacturing Industry	3,460,301.0	702,891.6	2,331,292.6	6,494,485.2
D	Electricity and Gas Supply	6420.9	2535.2	852.2	9808.3
E	Water Supply, Waste Management, Waste and Recycling	5921.3	−4280.7	2260.7	3901.3
F	Construction	911,609.9	261,438.9	31,644.6	1,204,693.4
G	Wholesale and Retail Trade; Car and Motorcycle Repairing	1,666,458.7	155,891.4	898,924.7	2,721,274.8
H	Transportation and Warehousing	93,237.8	15,100.7	6200.1	114,538.6
I	Provision of Accommodation and Food and Drink	66,124.7	13,826.9	26,231.3	106,182.9
J	Information and Communication	72,738.5	97,296.0	119,856.0	289,890.5
K	Financial Services and Insurance	121,722.7	−6254.8	−43,999.1	71,468.8
L	Real Estate	125,203.2	22,817.0	−54,772.2	93,248.0
M,N	Company Services	898.3	809.3	471.2	2178.8
O	Government Administration, The National Defense, and Social Security	396,379.4	−113,255.0	27,169.2	310,293.6
P	Education Services	99,950.4	4350.1	−816.0	103,484.5
Q	Health Services and Social Activities	22,851.0	16,830.2	1350.8	41,032.1
R,S,T,U	Other services	62,679.4	68,676.6	12,985.4	144,341.4

**Table 3:** Shift share calculation results for manufacturing sector.

Description		ShiftShare (million IDR)			
		RS	PS	DS	SS
Manufacturing Industry		3,460,301.0	702,891.6	2,331,292.6	6,494,485.2
1	Coal and Oil and Gas Refinery Industry	–	–	–	–
2	Food and Beverage Industry	3,071,327.6	1,264,807.3	2,465,032.4	6,801,167.3
3	Tobacco Manufacturing	–	–	–	–
4	Textile and Apparel Industry	7987.1	−509.1	677.6	8155.7
5	Leather, Leather Goods, and Footwear Industry	0.0	0.0	0.0	0.0
6	Industry of wood, goods made of wood and cork, and woven goods from bamboo, rattan, and others	12,559.4	−2546.3	−261.6	9751.5
7	Paper and Paper Goods Industry, Printing and Recording Media Reproduction	18,184.4	−11,477.8	3730.2	10,436.8
8	Chemical, Pharmaceutical, and Traditional Medicine Industry	13,049.0	14,768.4	−13,376.8	14,440.5
9	Rubber Industry, Rubber and Plastic Goods	307,261.1	54,049.9	−745,546.6	−384,235.6
10	Non-Metal Mineral Industry	23,523.5	3225.3	889.1	27,637.9
11	Base Metal Industry	0.0	0.0	0.0	0.0
12	Metal, Computer, Electronic, Optical, and Electrical Equipment Industry	3516.6	1262.4	65.7	4844.7
13	Other Machinery and Equipment Industry	–	–	–	
14	Transport Equipment Industry	1620.5	−916.6	12.6	716.6
15	Furniture Industry	923.0	132.0	83.9	1139.0
16	Other processing industry, repair, and installation of machinery and equipment	348.7	−11.9	94.0	430.7

The GRM method is used to identify potential economic sectors based on GRDP growth criteria (competitive advantage). In the method, a regional sector's growth is compared to a global area, both on a large and small scale. The results showed that the manufacturing industry category can grow in Bengkalis and Riau (Table 4). This is following the results of previous research, that the manufacturing industry is a serious concern to be further developed as it has a structure change value of 0.78 %. Among the eight other sectors experiencing similar conditions, the manufacturing industry was classified as the dominant [30,31]. The results of the GRM analysis in the manufacturing industry as in Table 5 showed that several sub-categories also became dominant sectors in line with their categories. These categories include 1) the Food and Beverage Industry, 2) the Chemical, Pharmaceutical, and Traditional Medicine industry, 3) the Non-Metal Mineral Industry 4) the Metal Goods Industry, 5) the Computers, Electronic Goods, Optics, and Electrical Equipment industry, 6) Furniture Industry, and 7) Other Manufacturing Industry.

**Table 4:** Calculation results of growth ratio model (GRM).

Category	Description	Riau Growth Ratio		Bengkalis Growth Ratio		Note
		Real	Value	Real	Value	
A	Agriculture, Forestry, and Fisheries	0.9	–	0.9	–	
B	Mining and excavation	0.1	–	0.8	–	
C	Manufacturing Industry	1.1	+	1.3	+	Dominant
D	Electricity and Gas Supply	1.2	+	1.2	+	Dominant
E	Water Supply, Waste Management, Waste and Recycling	0.7	–	0.8	–	
F	Construction	1.1	+	1.1	+	Dominant
G	Wholesale and Retail Trade; Car and Motorcycle Repairing	1	–	1.2	+	Potential
H	Transportation and Warehousing	1.1	+	1.1	+	Dominant
I	Provision of Accommodation and Food and Drink	1.1	+	1.2	+	Dominant
J	Information and Communication	1.6	+	2.2	+	Dominant
K	Financial Services and Insurance	1	–	0.8	–	Potential
L	Real Estate	1.1	+	0.9	–	
M, N	Company Services	1.4	+	1.5	+	Dominant
O	Government Administration, The National Defense, and Social Security	0.9	–	0.9	–	
P	Education Services	1	–	1	–	
Q	Health Services and Social Activities	1.3	+	1.3	+	Dominant
R,S,T,U	Other services	1.5	+	1.5	+	Dominant

**Table 5:** GRM calculation results for manufacturing sector.

Description		Riau Growth Ratio		Bengkalis Growth Ratio		Note
		Real	Value	Real	Value	
Manufacturing Industry		1.1	+	1.3	+	Dominant
1	Coal and Oil and Gas Refinery Industry					
2	Food and Beverage Industry	1.2	+	1.5	+	Dominant
3	Tobacco Manufacturing					
4	Textile and Apparel Industry	1.0	–	1.0	–	
5	Leather, Leather Goods, and Footwear Industry					
6	Industry of wood, goods made of wood and cork, and woven goods from bamboo, rattan, and others	0.9	–	0.9	–	
7	Paper and Paper Goods Industry, Printing and Recording Media Reproduction	0.7	–	0.8	–	
8	Chemical, Pharmaceutical, and Traditional Medicine Industry	1.5	+	1.0	+	Dominant
9	Rubber Industry, Rubber and Plastic Goods	1.1	+	0.0	–	
10	Non-Metal Mineral Industry	1.1	+	1.0	+	Dominant
11	Base Metal Industry					
12	Metal, Computer, Electronic, Optical, and Electrical Equipment Industry	1.2	+	1.1	+	Dominant
13	Other Machinery and Equipment Industry					
14	Transport Equipment Industry	0.8	–	0.7	–	
15	Furniture Industry	1.1	+	1.1	+	Dominant
16	Other processing industry, repair, and installation of machinery and equipment	1.0	–	1.1	+	

### 3.2 The Sustainability Status Analysis of the oil palm manufacturing industry

The RAP-Multidimensional analysis produced a sustainability index value for the oil palm manufacturing industry which is viewed from three perspectives, namely the companies, the household, and the village development. On the companies perspective, there are 42 variables, which are categorized into five dimensions, namely 9 economics, 7 socials, 10 environmentals, 9 technologies, and 6 institutionals. The household side has 32 variables divided into four dimensions, which include 9 economics, 11 socials, 6 environmentals, and 6 institutionals. Meanwhile, the Village Development aspect has 122 variables and was grouped into five dimensions, namely 24 economics, 35 socials, 33 environmentals, 16 technologies, and 14 institutionals. Table 6 shows the sustainability status of the analysis results.

**Table 6:** Multi-dimensional sustainability index and status.

Side	Index	Status
Companies	59.76	Sufficiently Sustainable
Household	60.16	Sufficiently Sustainable
Village Development	57.60	Sufficiently Sustainable

The multi-dimensional sustainability index values from the household, village development, Companies perspective were 60.16, 57.60, and 59.76, respectively. Therefore, they were all classified as sufficiently sustainable. In addition to analyzing the multi-dimensional sustainability status, the RAP analysis was also reviewed based on each dimension. To determine the variables affecting the oil palm manufacturing industry's sustainability in Bengkalis Regency, leverage analysis was performed.

**Fig. 4:**
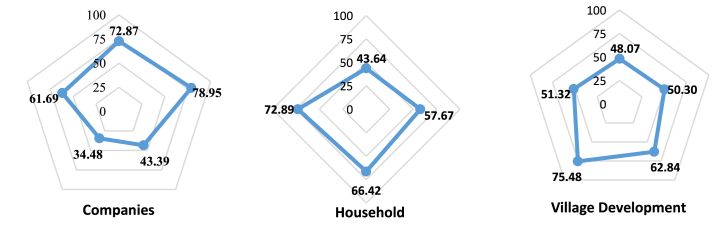
Multi-dimensional sustainability index diagram.

Fig. 4 shows the sustainability index value for each dimension. Judging from the technological dimension, the company has the lowest score of 34.48 and the environmental score of 43.39 so both are included in the less sustainable category. Meanwhile, the social dimension achieved the highest value of 78.95 and was classified as very sustainable. The economic and institutional dimensions were considered sufficiently sustainable since the index value ranges from 50.01 to 75.00. This is in accordance with the results of previous research, that oil palm and its derivatives caused economic multiplier effects in rural areas, particularly in the plantation [32]. Research results from several palm oil producing countries found that palm oil contributes to reducing poverty significantly, such as Indonesia [33], Ghana [34], Colombia [35]. The results of this study reveal that poor households in rural areas gain the most from oil palm farming. This proves that palm oil activities can reduce poverty in rural areas. However, from the other side, land clearing for oil palm has occurred as land conversion from production forest to oil palm plantation area [36].

**Fig. 5:**
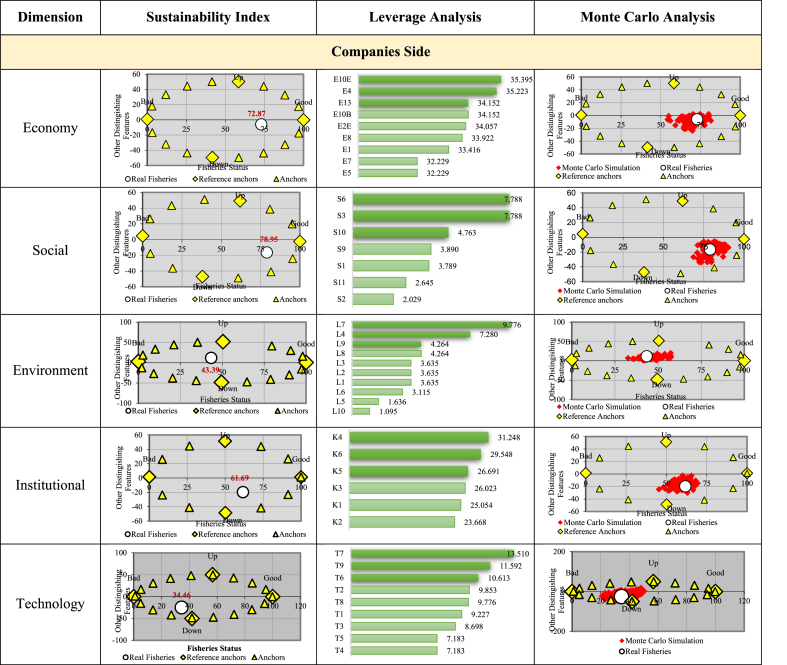
Sustainability index status, leverage analysis, and **Monte Carlo analysis on companies side**.

The sustainability value, which was quite high in the economic dimension was influenced by several variables with fairly high scores. It was observed that the industry's greatest optimism about prospects for oil palm was improving. One of the most sensitive variables is the domestic capital ownership proportion, which is higher than foreign ownership. The oil palm industry in Bengkalis Regency has impacted the surrounding community's social aspects by being responsible for workers and the surrounding community. This is evidenced by the high value of the social dimension index. The rural development sustainability index is presented in Fig. 5.

The environmental dimension showed a 43.39 value with a less sustainable status, caused by the high demand for oil palm raw materials, both from independent smallholders and plasma plantation, thereby leading to new land clearing. Moreover, very few companies applied sustainable principles, which is the reason Indonesian Sustainable Palm Oil (ISPO) is required in order to have a standard industry [[37], [38], [39]].In both the plantations, the system showed that many institutions have roles regarding input, finance, market, land, information technology, and institutional accesses. The sustainability analysis of the oil palm industry showed a score of 61.69, indicating a moderately sustainable status. In the technology dimension, the value was 34.48 and classified as very low or less sustainable. This is because very few CPO companies are developing derivative products, with none refining or processing the CPO into finished goods. Similarly, no single CPO industry in Bengkalis was producing biodiesel, despite the very high demand for biofuels domestically.

Based on the leverage analytical results shown in Fig. 5, the following sensitive variables affected the sustainability index value of each dimension.

**Table:** 

Economic Dimension	1.	percentage of companies with 100% domestic equity ownership
	2.	percentage of companies with the perception that oil palm prospects tend to be better in the future
	3.	the increasing or constant percentage of companies that perceive the change in profit compared to the previous year
Social Dimension	1.	percentage of companies having community empowerment programs
	2.	percentage of ‘s workers with health insurance
	3.	percentage of companies having health facilities
Environmental	1.	percentage of companies having waste treatment
Dimension	2.	percentage of companies that do not know how to implement environmental management
	3.	percentage of companies using renewable energy sources
Institutional Dimension	1.	percentage of companies that have or join a labor union
	2.	percentage of companies partnering with other companies
	3.	percentage of companies that have developed a farmer group
Technology Dimension	1.	percentage of companies having CPO quality standards
	2.	percentage of companies having waste treatment technology
	3.	number of boilers owned and used by the companies

**Fig. 6:**
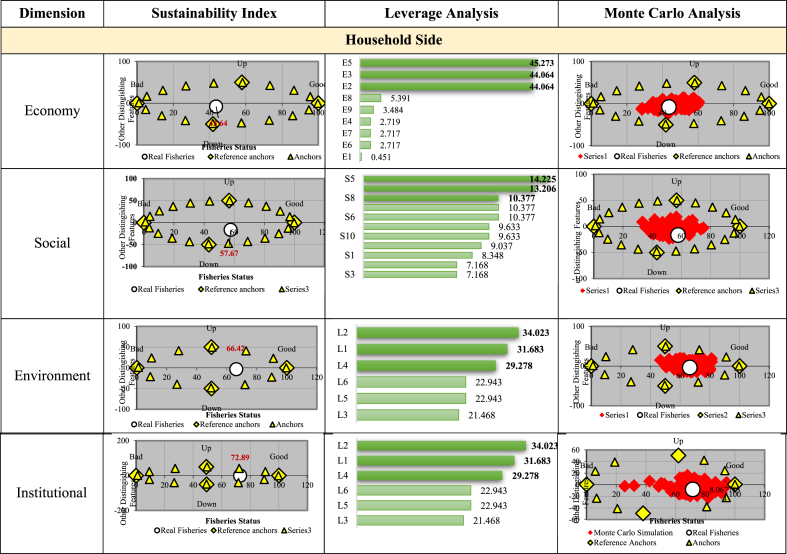
Sustainability index status, leverage analysis, and Monte Carlo analysis on household side.

In the Household perspective shown in Fig. 6, the lowest sustainability index score for each dimension was the economy with a value of 43.64, followed by the Social having 57.67. The environmental dimension with a 66.42 score was included in the sufficiently sustainable category, while the institution had the highest score of 72.89 and was categorized as very sustainable. The diversity of household livelihoods showed that the local community also works as planters or employees in the oil palm industry. Household optimism in assessing prospects for oil palm was dominated only by smallholder households. Meanwhile, non-oil palm smallholder households were not too optimistic about the prospects. The same applies to the household economic conditions/conditions when compared to the previous year. The farmer's households reported that it had increased but the non-oil palm farmers stated it was still the same and even decreased.

Many studies have been conducted in Indonesia and Malaysia showing that oil palm farming contributes to the regional economy and increases the income of rural communities. Palm oil activities provide financial resources which have an impact on increasing the amount of money circulating in rural areas. This condition causes an increase in the purchasing power of rural communities. Synergy also has an impact on investment to encourage the expansion of commercial agriculture [[40], [41], [42]]. Other research reveals that oil palm farming provides decent profits, contributing to regional consumption growth and the expansion of the non-agricultural sector [43].

The oil palm industry in Bengkalis Regency affected the community's social aspects. This result is in line with the Timbalino, that the ‘s responsibility towards the community and the environment is an integral part of the their activities. The oil palm industry's efforts were executed to create a more positive social impact on the surrounding community. This is evidenced by the increasing value of the Social Dimension indeks [34]. The existence of oil palm plantations affected the surrounding environment. For example, the clearing of new land often causes flooding due to reduced water absorption area. Also, the waste generated by the industry has the potential to cause losses to the community, particularly when not managed properly and appropriately. The increasing volume of palm oil waste has pollution implications and needs to be addressed. Especially for households living about 1–2 km from the industry [44]. It was observed from the institutional dimension that the demand for sustainable financing is increasing. Banks seek to contribute positively to the environmental and social issues occurring in the oil palm sector by requiring their customers to comply with sustainability standards.

**Fig. 7:**
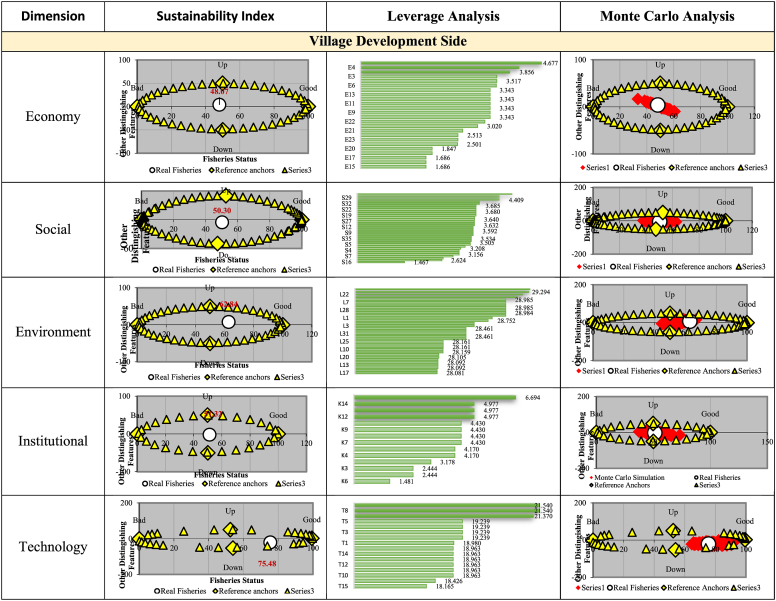
Sustainability index status, leverage analysis, and Monte Carlo Analisis analysis on village development side.

From the Village Development shown in Fig. 7, the sustainability index value for the economic dimension was 48.07, hence, it was categorized as less sustainable. Furthermore, the Social, Institutional, and environmental dimension have the respective values of 50.30, 51.32, and 52.84, hence they are considered moderately sustainable. The technology dimension has the highest index value of 75.48 with a very sustainable status.

Based on the leverage analysis results from the household perspective, the following sensitive variables affect the sustainability index value of each dimension.

**Table:** 

Economic Dimension	1. there are changes in profit or it increased compared to the previous year 2. total FFB production 3. income value
Social Dimension	1. social benefits due to the CPO industry 2. benefits of infrastructure development due to there are the CPO industry 3. there are workers' health insurance
Environmental Dimension	1. the factory waste disturbs households 2. the distance of the oil palm industry from people's residence 3. environmental-related conflicts between communities and the CPO industry
Institutional Dimension	1. source of financing in the oil palm business 2. difficulty in obtaining credit facilities for the oil palm business 3. obtaining credit from financial institutions

It is important to reiterate that the oil palm industry's sustainability value in village development was still relatively low in the economic dimension (48.07). This is characterized by the lack of economic facilities or infrastructure, such as markets, shops, supermarkets, grocery stores, and food stalls. In addition to the economic facility's absence, this low level was also observed in the Village Unit Cooperatives, Savings and Loans Cooperatives, as well as other cooperatives in the village. The oil palm industry in Bengkalis Regency affected both the ‘s social aspect in the community and also the village's condition. One of their duties was to take social responsibility regarding the community and the environmental conditions.

The oil palm industry sustainability index for the environmental dimension shows a fairly sustainable status with a value of 62.84. In general, the villagers assumed that the waste produced was not too disturbing and does not cause pollution. Meanwhile, its impact was only felt by people living close to the industrial locations. The village officials also stated that water pollution does not occur in the river flowing through the village. In the aspect of soil pollution, several companies performed waste management using land application techniques. This was not considered a disturbance to the village's condition because the waste disposal was practiced in the plantation areas owned by the companies [45].

The government has made various efforts to reduce the digital gap between villages and cities through the Ministry of Communications and Information Technology. Based on the sustainability index analysis of the oil palm industry (CPO) in the villages, the technological dimension was 75.48. It was observed from the study location that 1) residents use mobile phones, 2) there are cafes, indicating internet access, 3) mobile phone signal is quite strong in most of the areas, and 4) radio as well as television programs were broadcasted in the villages. It is important to note that despite the Internet access, there are several offices in which the Internet does not work properly.

From the leverage analysis results of all dimensions, the following are sensitive variables affecting the sustainability index value of each dimension.

**Table:** 

Economic Dimension	1.	the economic condition of the village is better than a year ago
	2.	number of economic facilities/market
	3.	prospects for oil palm
Social Dimension	1.	there are conflicts between the community and the companies
	2.	The CPO industry provides social benefits for the community
	3.	there are village community empowerment programs
Environmental	1.	the presence of settlements on the river's bank
Dimension	2.	the condition of river is polluted with waste
	3.	Environmental pollution of the soil
Institutional Dimension	1.	there are groups of fostered farmers
	2.	there are cooperatives
	3.	village council activities
Technology Dimension	1.	there are internet facilities in the village office
	2.	some computers/laptops are still functioning in the village office
	3.	mobile phone signal in most village areas

The MDS goodness-of-fit is reflected in the value of S-Stress and R^2^ [46]. A good model is indicated by the S-Stress value below 0.25 or S < 0.25 and R^2^, which is close to 1. Table 7 shows that the S-Stress value was less than 0.25, while the R-Square was close to 1 or 100%. This means all dimensional analysis results from the perspective of the, household, and village development met the goodness-of-fit criteria and were feasible to be implemented. Similar results were recorded by studies that calculate goodness-of-fit using S-Stress and R-Square [47].

**Table 7:** S-stress and R-Square.

Dimension	Companies Side		Household Side		Village Development Side	
	S-Stress	R-square	S-Stress	R-square	S-Stress	R-square
Economy	0.00000	0.99988	0.00000	0.99987	0.00468	0.99888
Social	0.00126	0.99944	0.00000	0.99983	0.00400	0.99919
Environment	0.00045	0.99976	0.00137	0.99934	0.00341	0.99916
Institutional	0.00000	0.99986	0.00170	0.99944	0.00000	0.99975
Technology	0.00170	0.99944			0.00000	0.99977

### 3.3 Sustainable oil palm industry development strategy

The oil palm industry development analysis was performed from the viewpoint of prices, exports, and product development because it has very promising prospects. It is supported by the suitability potential, land availability, and productivity that can still increase as the downstream industry is growing. The strategy for accelerating industrial downstream is very suitable to increase added value. Based on the existing prospects and potential, the oil palm industry can be developed through empowerment in the upstream and downstream by involving the community, villages, government, etc [35]. To achieve development goals that are consistent with policy directions, a strategy is needed by observing and using leverage factors showing sensitivity and the sustainability status of each dimension. From the MDS analysis results, 18 leveraging factors were obtained as show in Table 8.

**Table 8:** The influence and dependence value of each factor.

Leverage Factor		Global Influence	Global Dependencies	Direct Influence	Direct Dependence
A	Domestic capital ownership	27	22	1.18	1.33
B	Prospects for oil palm	37	31	1.06	1.79
C	Changes in profit compared to the previous year	28	22	1.42	1.40
D	Community empowerment program	31	22	1.31	1.62
E	Workers' health insurance	7	18	0.22	0.17
F	Availability of health facilities	6	15	0.27	0.15
G	Having waste treatment	20	26	1.01	0.77
H	Implementing environmental management	27	28	1.15	1.18
I	Using renewable energy sources	25	23	1.18	1.16
J	Conflicts between communities and CPO manufacturing companies	29	22	1.12	1.47
K	Having CPO quality standards	25	25	1.22	1.11
L	Having waste treatment technology	28	26	1.20	1.29
M	Number of boilers owned and used	14	18	1.04	0.55
N	Join labor union	23	19	1.01	1.12
O	Partnering with other companies	19	26	0.83	0.71
P	Developing farmer groups.	16	18	1.18	0.67
Q	Sources of financing in agriculture	17	16	1.06	0.78
R	Receiving credit from financial institutions	17	19	1.42	0.72

**Fig. 8:**
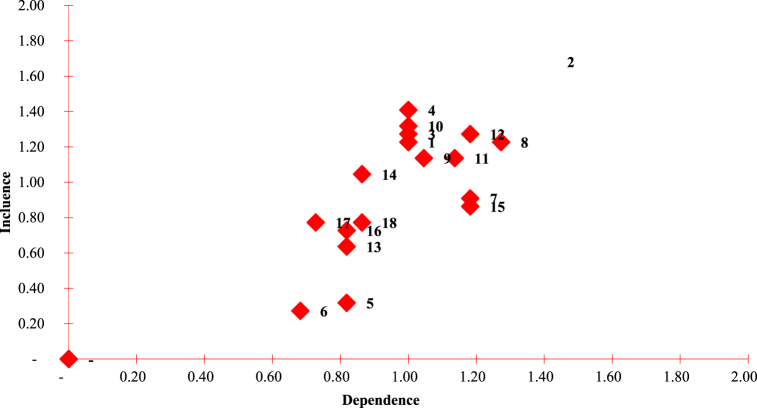
The influence factors importance level in the development of the oil palm manufacturing industry.

The identification results of the leveraging factors from the MDS analysis influencing the sustainable oil palm industry development according to the criteria considered were then conducted in a prospective analysis to determine the most important factors. The same research was carried out regarding prospective analysis to determine environmental management policy scenarios in industrial areas [48]. Involving local communities in decision making also fosters a sense of ownership and responsibility for the environment [49,50]. These leveraging factors were used as the basis for the expert assessment according to the prospective analysis questionnaire format. Specifically, the assessment was performed by twenty experts who understood and were directly involved in sustainable oil palm industry development. From the assessment results, a diagram of the influence and dependence factors was drawn to determine the key factors with the greatest influence. The factors contained in quadrants I and II were the factors that strongly affect ecovillage development. Based on the analysis results shown in Fig. 8. There are 10 main factors that are considered to influence sustainable development in the industry as show in Table 9.

**Table 9:** Important factors from prospective analysis results.

Dimension	Sustainability Index Value				Sustainability Status	Important factors of prospective analysis results
	Industry	Community	Village	Mean		
Economy	72.87	43.64	48.07	54.80	Sufficiently Sustainable	Domestic ownership of capital (A)
						Prospects for oil palm (B)
						Changes in profit compared to the previous year (C)
Social	78.95	57.67	50.30	62.31	Sufficiently Sustainable	Community Empowerment Program (D)
Environment	43.39	66.42	62.84	57.55	Sufficiently Sustainable	Applying environmental management (H)
						Using renewable energy sources (I)
						Environmental conflicts between communities and CPO manufacturing companies (J)
Technology	34.48	–	75.48	54.98	Sufficiently Sustainable	Having CPO quality standard (K)
						Having waste treatment technology (L)
Institutional	61.69	72.89	51.32	61.97	Sufficiently Sustainable	Joining/Having the labor union (N)
Multidimensional	58.24	60.15	57.60	58.32	Sufficiently Sustainable	

Furthermore, a policy scenario for the oil palm industry development was formulated based on these key factors as shown in Table 10. There are three possible future performances for each key factor, which include constant, increasing, or decreasing. Each key factor was coded A to J, while the possible conditions are numbered 1 (increasing), 2 (constant), and 3 (decreasing).

**Table 10:**
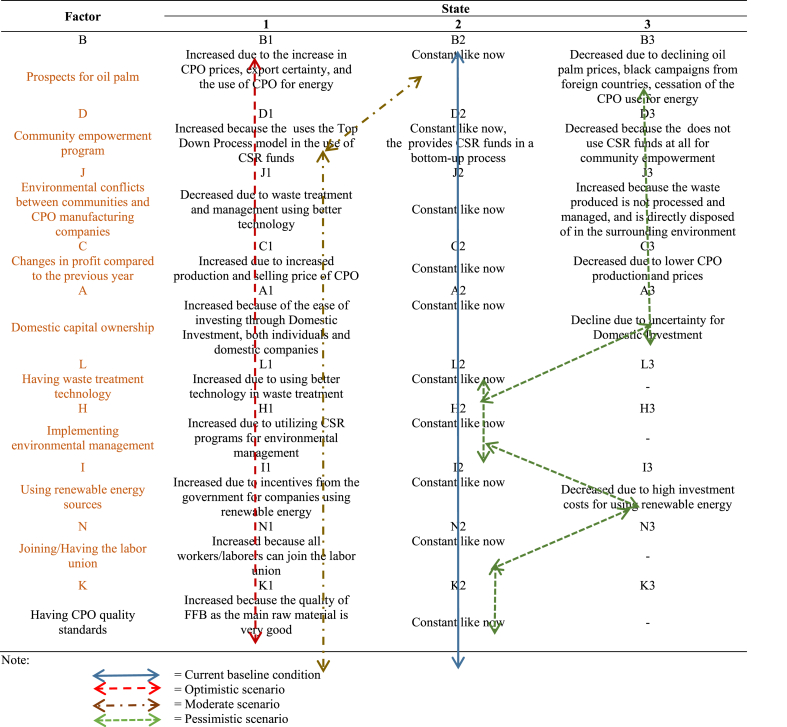
Mapping the determinants state of the Sustainable Oil Palm industry development.

The scenario preparation aims to predict the possibilities occurring in the key factors, such as whether the oil palm industry is developing, not changing (constant), or deteriorating [51]. In general, many possible scenarios can be generated but only three were created in this current study, namely pessimistic, moderate, and optimistic, as shown in Table 11. The pessimistic scenario is a condition that occurs when 1) the prospect of oil palm declines, 2) the waste utilization decreases, 3) the post-harvest product processing decreases, 4) the community empowerment programs decrease, 5) the environmental conflicts between communities and companies increase, 6) the changes in profits compared to the previous year decrease, and 7) the renewable energy usage decreases, while the other factors are static.

**Table 11:** The policy strategy scenarios for the development of a sustainable oil palm manufacturing industry.

Scenario	Condition
Current Condition (baseline)	B2 - D2 - J2 -C2 - A2 - L2 - H2 – I2 – N2 – K2
Optimistic	B1 - D1 - J1 -C1 - A1 - L1 - H1 – I1 – N1 – K1
Moderate	B2 - D1 - J1 -C1 - A1 - L1 - H1 – I1 – N1 – K1
Pessimistic	B3 - D3 - J3 -C3 - A3 - L2 - H2 – I3 – N2 – K2

The moderate scenario is a situation in which the prospect for oil palm does not change while other factors increase. Meanwhile, the optimistic scenario is a situation that occurs in line with the prediction and the government is expected to make efforts in improving the 10 important factors affecting the sustainable development. It is necessary to involve all parties in this scenario, starting from companies, communities, villages, as well as the regional and central government. These results are in accordance with previous findings, that industrial development depends on the government's role in building networks, mobilizing collective action, and triggering the development of a collective regional spirit [52]. The results of previous research, controlling palm oil waste with technology will have an impact on controlling the palm oil business in the long term. The utilization of technology reduces the risk of palm oil waste. Full-scale adoption of new technologies, best practices, and applied management concepts for the management of oil palm-derived products, including lessons learned from unsuccessful experiences [53].

In the long term, investment in palm oil development must focus on addressing social, ethical, ecological, economic and market problems [54]. Furthermore, sustainable investment does not only focus on the economy and financial markets but also employee performance as a valuable asset for the industry [55]. Oil palm plantation activities have significantly helped the economies of producing and non-producing countries by facilitating the export of crude palm oil and its by-products and creating jobs [56]. The successful development of oil palm plantations in Southeast Asia has made a real contribution to the economy of rural communities and poverty alleviation. Even though it has an impact on the environment [57]. Oil palm farming activities have accelerated the rural economy, reduced poverty in rural areas, and increased household food security in rural areas [7,8]. In order to maintain environmental sustainability, palm oil business players in Indonesia have implemented the concepts of the Roundtable on Sustainable Palm Oil (RSPO) and Indonesian Sustainable Palm Oil (ISPO). Each organization agrees to create and apply international standards to achieve sustainable palm oil planting and processing. This is done so that every palm oil production process is environmentally friendly.

## 4 Conclusion

The Shift Share and GRM analysis results underscore the high potential of the food and beverage industry, particularly in the context of the oil palm sector, due to its robust growth and competitiveness in Bengkalis and Riau. The sustainability assessment from different perspectives, including entrepreneurs, households, and village development, reveals a generally positive outlook, with notable strengths in the social and environmental dimensions.

The prospective analysis identifies ten pivotal factors crucial for the sustainable development of the oil palm industry, ranging from community empowerment to waste treatment technology. The recommended strategic scenario, an optimistic one, rightly emphasizes concerted efforts by the government to improve these key factors, requiring the active participation of stakeholders at all levels, from companies and communities to regional and central government bodies. This comprehensive assessment provides valuable insights for shaping the future of the oil palm industry in a sustainable and collaborative manner.

This research is not free from limitations, namely the number of responses is still very limited from both companies, communities and This research is not free from limitations, namely the number of responses is still very limited from both the company, community, and village officials. Apart from that, the information provided by respondents was not optimal due to differences in thoughts, assumptions, and understanding. It is hoped that these limitations can produce further, more comprehensive research so that scientific information can be the basis for decision-making regarding the development of a sustainable palm oil industry.

## Data availability statement

Data will be made available on request.

## CRediT authorship contribution statement

**Fitri Hariyanti:** Writing – review & editing, Writing – original draft, Visualization, Validation, Software, Methodology, Formal analysis, Data curation, Conceptualization. **Almasdi Syahza:** Writing – review & editing, Writing – original draft, Validation, Methodology, Funding acquisition, Formal analysis, Conceptualization. **Zulkarnain:** Writing – review & editing, Supervision, Data curation, Conceptualization, Writing – review & editing, Supervision, Data curation, Conceptualization. **Nofrizal:** Writing – review & editing, Validation, Formal analysis, Conceptualization, Writing – review & editing, Validation, Formal analysis, Conceptualization.

## Declaration of competing interest

The authors declare that they have no known competing financial interests or personal relationships that could have appeared to influence the work reported in this paper.
